# Developing an Adolescent and Young Adult Oncology Program in a Medium-Sized Canadian Centre: Lessons Learned

**DOI:** 10.3390/curroncol31050181

**Published:** 2024-04-26

**Authors:** Ian Scott, Sapna Oberoi

**Affiliations:** 1 Department of Psychosocial Oncology, CancerCare Manitoba, Winnipeg, MB R3E 0V9, Canada; iscott@cancercare.mb.ca; 2 Paul Albrechtsen Research Institute, CancerCare Manitoba, Winnipeg, MB R2H 2A6, Canada; 3 Department of Pediatrics and Child Health, Max Rady College of Medicine, University of Manitoba, Winnipeg, MB R3A 1S1, Canada; 4 Department of Pediatric Hematology-Oncology, CancerCare Manitoba, Winnipeg, MB R3E 0V9, Canada

**Keywords:** adolescents and young adults with cancer, multidisciplinary oncology program, collaborative program development

## Abstract

The Adolescent and Young Adult (AYA) Program at CancerCare Manitoba (CCMB) has experienced tremendous growth since its inception. This report provides an overview of how the AYA program at CCMB was established and the crucial factors that led to its early accomplishments and continued expansion. These factors included actions and decisions made at the individual and organizational level that helped lay a strong foundation for the program’s sustained success. We hope that some of these lessons learned can be adapted and implemented by other oncology agencies to improve the care outcomes and experiences of AYAs living with cancer.

## 1. Introduction

In 2008, a Canadian National Task Force was formed to address the challenges facing adolescents and young adults (AYAs) with cancer. This effort was funded by the Canadian Partnership Against Cancer (CPAC) with the support of C17, a consortium of Canadian pediatric oncology centres. The Task Force supported the creation of Regional Action Partnerships (RAPs) across Canada in 2013, including in Manitoba [1]. Manitoba is the fifth most populous province, with an estimated 1.3 million people and only 3–4% of Canada’s population [2]. The Manitoba RAP is a multidisciplinary group that works to improve AYA care in Manitoba and implement initiatives according to nationally accepted principles for the optimal care of AYAs living with cancer. The Manitoba RAP includes AYA champions such as AYA patient partners, nurses, and physicians from pediatric and adult hematology/oncology and palliative care, specialists interested in psychosocial oncology, oncofertility, and survivorship care, and members of the clinical trial unit. Excellent articles on the history and chronology of AYA oncology in Canada have already been published in the literature [3,4].

The Manitoba RAP launched a concerted effort to establish a multidisciplinary AYA program at CancerCare Manitoba (CCMB), the provincially mandated agency providing clinical services for cancer and blood disorders for patients of all ages [5]. CCMB receives an average of 300 referrals per year of newly diagnosed AYAs (ages 15–39) with cancer. In 2016, the Manitoba RAP submitted a successful proposal to the CancerCare Manitoba Foundation (CCMF) to create a 1.0 FTE position for an AYA psychosocial oncology (AYA PSO) clinician. In 2023, CCMB expanded its multidisciplinary AYA oncology program to include four new dedicated AYA team members. The expansion consists of an AYA clinical nurse specialist, dietitian, program/research coordinator, occupational therapist, and administrative clerk. The program plans to create a pathway for patients to access AYA-specific physiotherapy services. In this commentary, we share the journey of how the AYA program in Manitoba grew into what it is today and discuss some of the critical factors that should be considered when developing an AYA program in a mid-sized centre. In doing so, we hope it could perhaps provide a roadmap to other cancer agencies looking to improve the care of AYAs living with cancer in their jurisdiction.

## 2. Important Early Steps in the Development of AYA program at CCMB

### 2.1. Selecting Priorities 

The aforementioned Canadian National Task Force for AYAs pinpointed six priority areas for improving AYA care: active therapy and supportive care, psychosocial needs, palliation and symptom management, survivorship, research/metrics, and awareness/advocacy [1,2,3]. While all six of these priority areas deserve attention in AYA programs, it is vital for any nascent program to narrow its focus to increase the likelihood of early success. Therefore, the Manitoba RAP members elected to focus on three objectives as a starting point. Those three areas included creating a psychosocial oncology (PSO) position dedicated to AYA care, addressing oncofertility through streamlining referral pathways and healthcare provider education and improving clinical trial access and accrual for AYAs. These priorities are frequently cited as key gaps to address in AYA program development across the globe [6,7,8,9]. In the local context, these priorities were chosen in part because a member of the Manitoba RAP already had expertise in psychosocial oncology, oncofertility, and clinical trials. Other cancer agencies may identify other priorities to focus on first when developing AYA programs, depending on their existing strengths and expertise.

It is important to note that the six original priority areas outlined by the Task Force have since received more attention and continue to be pillars undergirding the overall development of the AYA program in Manitoba. However, strategically selecting only three focus areas allowed the Manitoba RAP to set realistic goals and achieve some early success that could be built upon for future growth.

### 2.2. Funding and Cancer Organization Support 

As the Manitoba RAP ramped up its work between 2013 and 2016, a pivotal moment was the prioritizing of AYA care in the Manitoba Cancer Plan 2016–2021, which served as the overall strategic plan for CCMB. The Manitoba Cancer Plan identified AYAs with cancer as an underserved population and made it an organizational objective to develop “a new multidisciplinary care program for adolescents and young adults” [10] (p. 68). To some, simply referencing AYA care in a multifaceted 5-year strategic planning document may appear to be of questionable significance; however, there is no doubt it provided an excellent reference point and performance indicator as the RAP continued its work over the next five years to bring attention and services to this group of patients with unique needs. The lesson learned was that when AYA care becomes an explicit organization-wide priority, it becomes easier to garner support from all stakeholders, including senior leadership. Having the backing of the cancer agency’s leadership team and getting other stakeholders to champion the cause is a proven strategy in AYA program development [11,12]. Any new AYA program would be well served by connecting with key individuals and departments that oversee the development of organizational strategic plans to help make AYA care a shared goal within their cancer agency. 

## 3. Building Momentum in the AYA Program

### 3.1. Leveraging AYA-Specific Clinical Staff 

Any new AYA program trying to find its footing ultimately has to create AYA-specific position(s) and hire appropriate clinicians and then provide sufficient leeway so that those same clinicians can undertake the dynamic task of developing and augmenting AYA services. Those clinicians can come from a variety of professional disciplines; for instance, in Manitoba, it was a psychosocial clinician with a social work background who undertook this role initially. At the outset, the AYA PSO clinician conducted an environmental scan of the existing AYA resources locally, nationally, and internationally, including informative site visits to extant AYA programs at Princess Margaret Cancer Centre in Toronto, Ontario, and the Jewish General Hospital in Montreal, Québec. As counselling referrals started to increase throughout 2017, the AYA PSO clinician engaged in other collaborations to first and foremost improve the care and experiences of AYAs with cancer, as well as garner support from different stakeholders and raise the overall profile of the program in the province. For instance, the AYA PSO clinician partnered with CCMB’s Community Oncology Program to create a new AYA referral pathway for CCMB clinics alongside a comprehensive resource document for AYAs. The rollout of this new referral pathway and resource document involved speaking to every nursing and physician group at CCMB, which was an incredibly powerful method for bringing general awareness to the importance of AYA care. As a result, counselling referral rates to the AYA PSO clinician started to grow (Table 1). 

Another collaboration involved the AYA PSO clinician becoming a founding partner of CCMB’s Health Equity Collective, which is designed to educate, support, and challenge CCMB and its employees to address health equity issues. AYAs are commonly identified as an underserved population, and addressing issues related to equity and access has been a motivator for AYA program development in other countries as well [7]. With this involvement in the health equity collective, the AYA PSO clinician can help centre the concerns of equity-denied groups of AYAs, such as patients who identify as part of 2SLGBTQIA+ and BIPOC communities. Furthermore, the AYA program has recently completed a qualitative research study exploring the experiences of racially, ethnically, gender, or sexually diverse AYAs and identifying ways to improve their care within the Canadian healthcare system; the data from this study are currently being analyzed. 

The existing Young Adult Cancer Support (YACS) group at CCMB saw burgeoning growth as average attendance tripled between 2017 and 2022. This rapid growth was in part due to having a dedicated AYA PSO clinician leading the group who was able to recruit more group members, which provided further evidence of the value of AYA-specific clinicians. During this period, the AYA PSO clinician also offered more AYA-specific programs, responding to patient feedback about the desire for services tailored to this unique demographic. This entailed co-facilitating an AYA narrative therapy group and offering an AYA-only “Coping With Brain Fog” cognitive rehabilitation program [13], the latter of which was conducted as a feasibility and acceptability study.

### 3.2. Defining Age Cut-Off for AYA Oncology Services 

In North America, AYA programs tend to serve patients between the ages of 18 and 39. The AYA program at CCMB is currently available to 15–39-year-olds with cancer and their supporters, but that age range was arrived at incrementally, and it is worth examining how CCMB’s AYA program settled on that age spectrum, beginning with the upper end. For a short time, the program had an age restriction of 29 during the piloting phase of the program; however, the age eligibility for referral to the AYA PSO clinician was quickly raised to 39 to address the distinct needs of patients in their 30s who are still dealing with the challenges that make the AYA cancer experience different (e.g., work/school interruption, relationship stressors, social isolation, fertility, etc.). 

When considering that the AYA program’s minimum age cut-off of is 15, it is vital to consider the difficulties some AYA patients experience when transitioning from pediatric to adult care [14], so allowing the AYA PSO clinician to overlap pediatric and adult clinics was crucial. Effective collaboration across pediatric and adult oncology care providers has been demonstrated to be an important ingredient in AYA program development across other high-income countries, including the USA, Australia, and the United Kingdom [7,15]. The inclusion of the pediatric patient group also enabled the AYA PSO clinician to offer services to childhood cancer survivors receiving long-term follow-up care at CCMB who may have been treated as young children but were now between the ages of 15 and 39. These long-term childhood cancer survivors are often the forgotten demographic within AYAs with cancer, so reaching this group of patients should be a priority for other jurisdictions planning their program delivery. Notably, Manitoba has a distinct advantage in that pediatric and adult patients are all treated by CCMB, allowing for a lot of natural collaborative care between pediatric and adult providers, which may not be as accessible in other contexts. Nonetheless, all AYA programs should strive to serve an age range that matches other jurisdictions, but it may be wise to expand program eligibility in an incremental way.

### 3.3. Fostering National Collaborations 

In a Canadian healthcare system that is generally fragmented along provincial lines, partnering with organizations outside of the province can tap into more opportunities for any program’s growth. For example, the AYA PSO clinician worked to foster robust partnerships with AYA organizations outside of CCMB. Young Adult Cancer Canada (YACC) formed half of one such partnership, which allowed both CCMB and YACC to reach a broader base of AYAs with cancer than would have otherwise been possible. For instance, the AYA PSO clinician routinely refers patients to YACC programming, and the social programming at YACC regularly opens doors for young adults seeking clinical services at CCMB. Treating this as a true partnership, rather than simply like-minded entities existing alongside each other, was yet another instrumental factor in the growth of CCMB’s AYA program. So much so that CCMB, the CCMF, and YACC collaborated to offer Primetown in 2021 and 2022, a large virtual summit for young adults with cancer and anyone who supports them, including their families and professional care providers. These online summits experienced overwhelming success, with 907 registrants in 2021 at the height of the COVID-19 pandemic and 682 registrants in 2022. This included registrants from every Canadian province and territory and 18 different countries. This raised the visibility of the CCMB AYA Program and YACC, as 39 different types of professionals from 106 unique health centres took part in the summit [16]. The AYA program at CCMB was also engaged with the Canadian Partnership Against Cancer’s (CPAC) AYA National Network during this time, providing input for the Canadian Framework for the Care and Support of AYAs with Cancer [17] and for ongoing oncofertility initiatives that will continue to expand in 2024. CCMB looks forward to contributing to more national initiatives that will lead to more coordinated and comprehensive care for AYAs, since a nationwide approach has proven to be successful elsewhere in the world, notably by the Australian Youth Cancer Services [18].

### 3.4. Enhancing Healthcare Provider Education and Awareness 

Aside from the daily clinical counselling and the aforementioned collaborative efforts, the AYA PSO clinician continued to provide regular consultation and education to colleagues regarding the distinct needs of AYA oncology patients. This allowed the AYA program to identify other AYA “champions” working in different areas of CCMB, who could then take on the informal role of promoting the importance of AYA care within their respective professional disease site groups. An important lesson to learn in this process for any emerging AYA program is to always be looking outside itself to develop AYA awareness and expertise on a larger scale. This collaborative and consultative approach enabled the AYA program to have a genuinely provincial mandate, helping AYAs with cancer connect to counselling, support groups, and educational opportunities across Manitoba. Fostering this connection with AYAs in different regions of the province dovetailed nicely with the transition to virtual counselling and programming necessitated by the onset of the COVID-19 pandemic in March 2020.

## 4. Further Expansion of the AYA Program 

### 4.1. Identifying the Current Unmet Needs of AYAs 

To better identify the present unmet needs of AYAs in Manitoba, the AYA PSO clinician and the AYA program lead conducted two focus groups in the summer of 2021 with diverse AYAs with lived experience of cancer treated at CCMB (unpublished data). The most common themes of unmet needs emerging from these focus groups included reducing delay in diagnosis, addressing AYA-specific information and supportive care needs at the time of diagnosis, access to AYA-specific supportive care programs such as physiotherapy, vocational rehabilitation, and a dietician, addressing oncofertility needs, improving access to clinical trials, and enhancing transition and survivorship care. These instructive focus group sessions with AYAs provided the insight and direction necessary to formulate a new proposal for program expansion that would lead to substantial growth and establish a truly multidisciplinary AYA program. Focusing on the experiences and voices of AYA patients and allowing that to guide AYA program development has been a successful strategy in other jurisdictions, and CCMB is following this patient-centred approach [11,19]. It should come as no surprise that there is tremendous value in authentically engaging AYAs themselves to help formulate the goals and future directions of an AYA program. 

### 4.2. Acquisition of New Funding for Developing a Multidisciplinary AYA Program 

Despite early successes in the first few years, the AYA team at CCMB continued to circle back to the aforementioned Manitoba Cancer Plan, the organization’s 2016–2021 strategic planning document. The goal of developing a truly multidisciplinary team had not yet been accomplished, and an initial attempt at further expansion in 2018 did not come to fruition. The lesson here is that program creation and expansion often do not take hold on a first attempt. Other AYA programs have experienced similar obstacles, where the multidisciplinary team can only be fleshed out over time, not all at once [11,20]. AYA programs everywhere can double back on their efforts, modify their approach, and continue advocating for increased AYA resources. 

Based on the original Canadian Task Force recommendations mentioned at the beginning of this report, the identified unmet needs of the patients from focus groups, stakeholder engagement, and a review of other established AYA programs across the world, we again sought funding from the CCMF in 2022 to further develop a multidisciplinary AYA Program at CCMB to strengthen, foster, and ensure clinical and research excellence. The expansion has the goal of providing personalized, multidisciplinary, and enhanced supportive care and education, access to clinical trials, psychosocial support, fertility preservation, sexuality counselling, physical and vocational rehabilitation, nutritional counselling, and palliative and survivorship care to AYAs diagnosed with cancer in Manitoba, as well as to establish a sustainable AYA research platform at CCMB to inform clinical care. This infrastructure grant application was accepted and generously funded for three years by the CCMF in alignment with CCMB’s strategic priority of improving AYA care and included funding for a full-time AYA clinical nurse specialist (CNS), AYA dietician, AYA physiotherapist, AYA occupational therapist, AYA program/research coordinator, and AYA administrative clerk. 

At the time of writing this report, we have hired the CNS, dietician, program/research coordinator, occupational therapist, and administrative clerk. The AYA CNS provides patient counselling and support from diagnosis to the survivorship period and has started partnering with each disease site group (DSG) to create seamless care experiences for AYAs. This involves providing the DSG staff with the knowledge, tools, capacity, and expertise to offer frontline support to AYAs for psychosocial, diet and nutrition, exercise, cancer education, sexuality, fertility preservation, and financial, work, and school concerns. With time, besides creating educational materials/resources for AYA patients and their supporters, the AYA CNS will develop educational modules for healthcare providers to increase awareness about the unique needs of AYAs with cancer. The CNS will advance knowledge of various stakeholders through formal and informal channels, including presentations at local, regional, and national levels. The AYA dietitian and occupational therapist have started providing individualized expertise to AYA oncology patients throughout the cancer trajectory. The program and research coordinator is helping in engaging patient partners in the AYA program’s development, creating educational resources for patients and their supporters, evaluating AYA program expansion, and enhancing the AYA research platform at CCMB. The AYA program is also working with the Community Oncology Program at CCMB to create a diverse patient partner engagement program, which will help co-design, create, and evaluate future AYA initiatives. It is true for any AYA program, and any cancer agency in general, that developing meaningful patient partnerships can significantly enhance the effectiveness and responsiveness of any initiative that is undertaken. It is equally true that for any AYA program, either in Canada or abroad, sustaining growth and progress requires ongoing reflection and evaluation [21]. 

## 5. Conclusions

While there are still many ways to improve the AYA program at CCMB in the years ahead, the growth of the AYA program at CCMB is quite an exciting development that can hopefully be used as a sort of blueprint in other jurisdictions across Canada and worldwide. While different challenges and opportunities will inevitably present themselves, we have learned that growing a robust AYA program will require a certain mix of foresight, organization-wide priority setting, financial support, patient engagement, dedicated staff, advocacy, persistence, and, perhaps most importantly, a collaborative approach. 

## Figures and Tables

**Table 1:** Number of AYA referrals seen by AYA psychosocial clinician (PSO) every year.

Year	Total AYA PSO Clinician Referrals
2017 ^1^	122
2018	177
2019	195
2020 ^2^	148
2021	186
2022	205

^1^ Referrals were only accepted as of 30 January 2017; ^2^ decrease in referrals during COVID-19 pandemic lockdown.
